# No Ifs, No Butts: Compliance with Smoking Cessation in Secondary Care Guidance (NICE PH48) by Providers of Cancer Therapies (Radiotherapy and Chemotherapy) in the UK

**DOI:** 10.3390/ijerph13121244

**Published:** 2016-12-15

**Authors:** Daniel Hutton, Ivan Gee, Ciara E. McGee, Rebecca Mellor

**Affiliations:** 1 Transforming Cancer Care Project, the Clatterbridge Cancer Centre NHS FT, Wirral CH63 4JY, UK; 2 Public Health Institute, Liverpool John Moores University, Liverpool L3 2ET, UK; I.L.Gee@ljmu.ac.uk (I.G.); mcgeeciara@yahoo.co.uk (C.E.M.); 3 Public Health Wirral Borough Council, Wirral CH62 3QX, UK; rebeccamellor@wirral.gov.uk

**Keywords:** smoking cessation, tobacco control, cancer care, NICE guidance

## Abstract

**Background**: Legislation preventing smoking in public places was introduced in England in July 2007. Since then, smoke-free policies have been extended to the majority of hospitals including those providing cancer therapies. Whilst studies have been conducted on the impact and effectiveness of hospital smoke-free policy in the UK and other countries, there have not been any studies with a focus on cancer care providers. Cancer patients are a priority group for smoking cessation and support and this study aimed to examine implementation of the National Institute Clinical Excellence (NICE) guidance (PH48) in acute cancer care trusts in the UK. **Methods**: Participants were recruited from UK radiotherapy and chemotherapy departments (total 80 sites, 65 organisations) and asked to complete a 15 min online questionnaire exploring the implementation of NICE guidance at their hospital site. **Results:** Considerable variability in implementation of the NICE guidance was observed. A total of 79.1% trusts were smoke-free in theory; however, only 18.6% were described as smoke-free in practice. Areas of improvement were identified in information and support for patients and staff including in Nicotine Replacement Therapy (NRT) provision, staff training and clarity on e-cigarette policies. **Conclusions:** While some trusts have effective smoke-free policies and provide valuable cessation support services for patients, improvements are required to ensure that all sites fully adopt the NICE guidance.

## 1. Introduction

Cigarette smoking remains a significant public health challenge. While rates of smoking have continued to decline over the past decades, around 20 percent of adults in England still smoke [1]. The smoking prevalence in the population of cancer patients rises to 31%, with 51% being ex-smokers [2]. Smoking is a significant aetiological factor in the development of a number of chronic illnesses including a range of cancers [3]. Cigarette smoking (both active smoking and second-hand tobacco smoke) causes almost a fifth (19%) of all cancer cases in the UK [4], with one in four cancer deaths attributable to cigarette smoking [5]. The Five Year Forward View [6] states that the sustainability of the National Health Service (NHS) now requires “a radical upgrade in prevention and public health”. The Achieving world class cancer outcomes [7] document echoes this sentiment, advocating the importance of secondary prevention in the oncology setting. The biggest avoidable risk factor associated with the development of a malignancy is smoking [8], consequently smoking cessation services play a key part. These services are both effective and cost efficient [9,10], and should form a routine element of health care provision [11].

Legislation prohibiting smoking in the workplace and enclosed public places was introduced in England in July 2007 as a consequence of the Health Act 2006 [12]. Since the implementation of smoke-free legislation, many NHS secondary care settings adopted smoke-free policies for enclosed areas and grounds [13]. This stance was further supported in 2011 by the Government Public Health White Paper Healthy Lives, Healthy People [14], which promotes smoking cessation through secondary care providers to reduce the burden of disease attributable to cigarette smoking. Previous studies have shown the implementation of smoke-free policies in both acute and mental health hospital settings is achievable [13,15] with potentially beneficial impacts on aspects such as exposure to environmental tobacco smoke [13]. Nevertheless, there have been reported problems with the engagement, compliance, and enforcement of smoke-free policies in hospital settings [16,17]. Therefore, in 2013, the National Institute for Clinical Excellence (NICE) published new guidance (PH48) to support smoking cessation, temporary abstinence from smoking, and smoke-free policies in all secondary care settings [18]. 

Secondary care settings can serve as an important platform to deliver smoking cessation advice [19]. Health care professionals such as therapeutic radiographers, allied health professionals and nurses can be important role models to promote smoke-free norms and behaviours to support the survivorship agenda in the oncology arena [20]. For over 10 years, NICE has recommended that health professionals actively identify smokers and refer them to appropriate support services [21]. Evidence supports the health professional’s role as a smoking cessation practitioner, as a prompt from a health professional is the second most common reason for a quit attempt [22]. Studies show that between 50% and 83% of cancer patients who smoke continue to do so during their treatment and beyond [23,24,25]. A recently published systematic literature review [26] highlights the significant benefit of patients stopping or abstaining from smoking during periods of treatment such as chemotherapy and radiotherapy. Stopping smoking, or even abstaining during treatments, can reduce acute treatment related effects [27], improve effectiveness of treatment [28] and reduce the probability of a second primary cancer [29] and disease recurrence [30]. Targeted cessation efforts during cancer treatments are thus integral to reduce acute sequelae and increase the effectiveness of treatment. Therefore, the aim of the present study is to explore UK Radiotherapy and Chemotherapy departments’ compliance with NICE Smoking Cessation Guidance (PH48) [18].

## 2. Materials and Methods 

### 2.1. Participants and Procedures

In 2015, a cross-sectional study was conducted to explore compliance with the NICE Smoking Cessation Guidance (PH48) [18] across all UK radiotherapy and chemotherapy departments (Total 80 sites, 65 organisations). Participants were recruited for the study using email invitations sent to all department managers via the Society and College of Radiographer’s mailing list. The email directed managers to the study, and targeted email invitations to forward to appropriate colleagues with a working knowledge of the smoke-free policy within their department. This study was conducted as an audit and the UK Research Ethics Committee deemed full ethical approval to be unnecessary. The questionnaire was completed using a web-based survey (www.surveymonkey.com). The online survey was designed to take no longer than 15 min to complete. Completed surveys were submitted by each participant, and responses immediately transmitted to a secure electronic database for subsequent analysis.

### 2.2. Measures 

A questionnaire using both open-ended and closed-ended questions was constructed for the purpose of this study. Using open-ended questionnaire items added richness and depth to quantitative data collated. Questionnaire items were developed in consultation with the research team following a thorough review of the NICE Guidance (PH48) [18].

The draft questionnaire was piloted with five respondents internally at the author’s trust. This allowed valuable information to be gained such as average time for completion (15 min), if the questionnaire was fully understood and if it delivered useable data. The questionnaire was reviewed in light of the pilot, and no significant amendments or changes were deemed necessary.

### 2.3. Analyses 

The frequency of response for each question was determined and tabulated in Microsoft Excel. Systematic data analysis consisted of examining the free text responses, then categorising, tabulating and recombining the data into themes. 

## 3. Results

### 3.1. Smoke-Free Policy 

Of the 43 NHS hospital trusts (66% participation rate) that participated in the research study, the majority (79.1%) were smoke-free in theory. Smoke-free in theory refers to a situation where a smoke-free policy is in place, although it is not adhered to. However, a relatively small proportion were described as being smoke-free in practice (18.6%), and one hospital had no imminent plans to become smoke-free (2.3%) (Table 1). All but one oncology department in each participating hospital currently has a smoke-free policy in place, with over half (52.4%) reporting that the policy is enforced.

Participants noted a number of enablers to achieving the smoke-free policy in practice, including having designated tobacco control staff, displaying smoke-free signage and electronic messages, and designated smoking areas, and offering smoking cessation support and disciplinary action for staff breaches (see Table 2). Several barriers in achieving the smoke-free policy in theory were also noted and included the lack of policy enforcement. Staff also reported concerns about the repercussions of enforcing the policy among patients and visitors, and patients’ choice to smoke (see Table 3).

Participants were also asked if electronic cigarettes were included in their hospital’s smoke-free policy. Of those who responded, 60.5% reported that they were and that their use was prohibited. Only one policy made any distinction between conventional cigarettes and electronic devices. Operationally, two departments allowed e-cigarettes to be used within the hospital if “used discreetly”. A further two trusts allowed e-cigarettes to be used outside, on hospital grounds. The reservation around the use of e-cigarettes included; the fire risk related to the device and chargers; unlicensed product with limited information admission that “policy has not caught up with e-cigarettes yet”.

Less than a third (32.5%) reported to “always” provide written and/or verbal information and advice about their smoke-free policy to patients (Table 4). Almost all hospitals (97.7%) reported that display posters and/or signage are used to convey smoke-free messages, with nearly a third (32.6%) using staff patrol and/or recorded messages at key locations within the hospital.

### 3.2. Information and Support for Patients

Participants were asked when the patient’s smoking status is investigated and whether it is revisited periodically during active treatment. All participants who responded (*n* = 30) reported that patients’ smoking status is recorded at the initial consultation phase. Of those who responded (*n* = 38), less than a third (31.6%) reported that they revisit their patient’s smoking status during active treatment. Participants were also asked if they inform patients about the acute side effects associated with smoking whilst undergoing cancer therapies. Of those who responded (*n* = 37), the vast majority (97.3%) reported that they do inform patients about the side effects of smoking during cancer treatment. Moreover, nearly nine in ten (86.5%) stated that they warn patients about the reduced effectiveness of cancer treatments if they continue to smoke during treatment but less than a third (32.4%) advise/or encourage patients to abstain from smoking for a period of time prior to and after cancer treatments (Table 1).

### 3.3. Provision of Smoking Cessation Support Including Pharmacotherapies

Participants were asked about the type of smoking cessation support available to patients within their Trust. Of those who responded (*n* = 30), pharmacologic intervention (36.7%) was the most frequent support available for patients, followed by individual (26.7%) and group (10.0%) behavioural support, with some providing “other” (20.0%) support and some offering no support (6.7%) to patients. Participants were asked whether patients are provided with information about the different types of smoking cessation pharmacotherapies and behavioural support available (Table 4). Pharmacotherapies are therapies employing pharmaceutical drugs, as distinguished from therapy using radiation (radiation therapy), cytotoxic agents (chemotherapy) or movement (physical therapy). Of those who responded (*n* = 31), a small proportion (16.1%) stated that they “always” provide patients with information about the types of smoking cessation support available. Similarly, only a small proportion (11.5%) reported that they “always” offer nicotine-containing products to patients who smoke and/or “always” (7.1%) encourage the use of licensed nicotine-containing products for anyone who does not want to completely quit smoking (Table 4). Smoking cessation advice was most often provided by a nurse or Clinical Nurse Specialist (CNS), followed by medics and the therapeutic radiographer.

### 3.4. Referral Systems for Smoking Cessation Support 

Participants were asked how much they agreed or disagreed with the statement “systems are in place for recording and maintaining records of patients smoking status”, using a five-point scale from strongly agree to strongly disagree. Of those who responded (*n* = 29), just over a quarter (27.6%) agreed (including agree/strongly agree) that systems are in place to record and monitor patient smoking status. 

Participants were also asked who provides patients with the first advice regarding stopping or reducing smoking. Of those who responded (*n* = 27), the majority stated that the medics (70.4%) are the first to give patients advice about smoking cessation followed by the CNS Nurse (11.1%) and the therapeutic radiographer (3.7), whilst others (14.8%) were unsure of who provided this advice to patients.

### 3.5. Training for Staff in Smoking Cessation 

Participants were asked about the type of training provided to front-line staff in smoking cessation services. Of those who responded (*n* = 27), only just over half (51.9%) reported that front-line staff receive very brief intervention advice/or advice level 1. The remainder (48.1%) received no training in smoking cessation (Table 1). 

### 3.6. Provision of Smoking Cessation Support for Employees

Participants were asked whether employee smoking cessation is included in their Trust’s policy and whether cessation support is available for staff who wish to quit smoking. Of those who responded (*n* = 27), the vast majority (85.2%) reported that employee smoking cessation is included in their hospital trust’s policy, with some respondents (*n* = 15) reporting that their Trust provides brief intervention support (including opportunistic advice, self-help materials, and or referral for more intensive support) to support employee smoking cessation.

## 4. Discussion

This study explored compliance with the NICE Smoking Cessation Guidance (PH48) [18] in Radiotherapy and Chemotherapy departments across the UK. Findings from this audit showed that whilst each oncology department had a smoke-free policy, just over half (52.4%) complied with the policy in practice. Although the majority of participants had reported to provide patients with information about the acute side effects of smoking during cancer treatments, less than a third (32.4%) advise patients to quit/abstain from smoking during cancer treatments. In addition, only a small proportion of participants offer nicotine-containing products to patients who smoke during treatment. Furthermore, respondents indicated specific concerns relating to the implementation of the smoke-free policy in oncology departments, especially with regard to policy enforcement, safety issues, and patient smoking norms.

It is clear from this audit that practice relating to smoking cessation support varied significantly from policy, with just over half (52.4%) complying with their policy. Deviation from policy is unlikely to be allowed in other areas of health care, such as standard operational procedures or infection control policies, and should not be accepted for smoking cessation. The discord between policy and practice reduces the overall effectiveness of any intervention. Additionally, it appears that policies do not reflect best practice, indicated by the evidence base and information detailed in the NICE guidance.

The main areas showing deviation from policy include smoking shelters, distinction between patients having treatment for radical or palliative intent, and facilitating non-ambulatory patients to smoke.

It is essential that when policies are developed and reviewed that the practical application is considered. Policies must be workable and sustainable with roles and responsibilities clearly stated. The policy requires buy-in from the individuals and teams expected to adhere to and deliver the policies, and the policy must be appropriately resourced by the organisation. For many trusts, the introduction of smoke-free policies started off positively, then later lost momentum.

Delivering smoking cessation messages needs to be done tactfully, with empathy in a non-judgmental manner. This is perhaps especially critical when considering patients with a cancer diagnosis. It is also important to realise that the life event of a cancer diagnosis has the potential to be a pivotal moment in an individual’s or family’s smoking behaviour. This “teachable moment” should not be wasted due to health professionals feeling uncomfortable or not competent to discuss and deal with the issue. Less than a third (31.6%) revisited a patient’s smoking status during treatment. Pattinson and Jessop [31] identified lack of knowledge or confidence as the significant barrier rather than a previous negative experience. Almost half (48.1%) of staff had no smoking cessation training. In this study, nurses were the main providers of smoking cessation advice and information (56.3%), followed by therapeutic radiographers (32%). The drive for Allied Health Professional (AHP) involvement in public health is a current priority of Public Health England (PHE) [32]. The developed strategy [33] seeks to build the capacity, impact and profile of AHPs in public health, with a key aim of equipping practitioners with the skills, knowledge and initiatives to promote the health and wellbeing of individuals. A recent study highlights that AHP’s are willing to accept this role, with over 86% of participants recognising that the prevention of ill health should be part of their role [34]. However, a separate study identified that only 35% of oncologists agreed they had the skills and expertise to deliver smoking cessation advice [35]. This is concordant with a sample of UK therapy radiographers, where 40% stated that they would be confident in delivering smoking cessation advice and interventions [31]. This study identifies that practitioners have concerns regarding offending patients and potentially compromising the therapeutic relationship. Similar practitioner perceptions have been reported by Pattinson and Jessop [31]. It is important that professionals are effectively trained in brief advice interventions and feel confident in using these techniques. Part of the role as a health professional is to provide patients with the best possible health care, and negating to provide smoking cessation advice and intervention, regardless of benevolent intention, contravenes this.

The reduced effectiveness of treatment in patients who continue to smoke, if communicated, was mainly communicated verbally (96%). The verbal message was supported by written information in less than a third of respondents. Given that the patient’s understanding may be compromised by anxiety, coupled with a lot of information, it is considered good practice to provide the message in multiple formats. Patients were informed of the increased acute side effects (97.3%) and the reduced effectiveness of treatment (86.5%) if they continued to smoke. Less than a third (31.6%) revisited smoking status during treatment, despite the fractionated nature of radiotherapy and chemotherapy lending itself to such an approach. Consistent repetition of the message throughout patients’ treatment with dialogue invited the patient to “check back” that they understand the message—in an emphatic approach.

The section relating to pharmaceutical interventions had a significantly lower response rate relevant to other sections. This indicates that smoking cessation services would benefit from a multidisciplinary approach to design and delivery.

Careful consideration needs to be given to the emerging evidence for the role of e-cigarettes as part of a harm reduction strategy. This is currently a live and controversial discussion, and a full analysis is beyond the scope of this paper. Some headliners for consideration are provided as follows: 

E-cigarettes are regulated in the UK under the Tobacco and Related Products Regulations [36], bringing tighter standards of safety and quality, and further restrictions on advertising and promotion. Manufacturers of e-cigarettes are required to conform to new safety standards, including specifying the ingredients used in their product and limiting the size of tanks and refills. While e-cigarettes are not currently available as licensed medicines, it is expected that products will come onto the market that can be prescribed on the NHS by General Practitioners and other health care professionals alongside other stop-smoking medicines once they are regulated as medicines [37]. Some resistance exists from smoking cessation personnel, with over half stating they would not recommend e-cigarettes to clients [38]. Although this may be a moot point, as practitioner–client interactions are driven by the client’s agenda and patients are using e-cigarettes now. Around 2.6 million adults in the UK use e-cigarettes [39], making it the most popular smoking cessation approach in England [40]. Therefore, clear guidance is required for practitioners. 

It is essential that patients motivated to stop smoking are not inadvertently directed towards a harm reduction approach, missing the opportunity for cessation support, as the evidence base indicates a quit attempt as best practice [41]. Stopping smoking, abstinence, or use of NRT sit on a spectrum, as does the use of smokeless products or vaping. The health community has previously been cautious to engage with the tobacco industry to develop “safer” products containing nicotine or tobacco. Reluctance and concern remains, although there is now an increasing evidence base [37,42,43,44,45,46,47] that vaping devices are a valuable tool in a multi-facetted approach to smoking cessation and harm reduction. 

It is important to have a fully congruent message on smoke-free sites; the signage and literature stating smoke-free trust can be undermined by the presence of cigarette butts on the floor or smoking furniture such as ash trays on top of bins. Some may see the acceptance of e-cigarettes as a blurred line, and this will need to be managed. Elements of the “broken window” theory [46], more commonly employed in criminology, may apply. Trusts need to establish and maintain that the expectation, reality, and norm is that it is a smoke-free site. This has been achieved, albeit with the considerable benefit of law and legislation, in the aviation industry. People do not smoke on long-haul flights as they know they cannot.

## 5. Limitations of Study

Questions in the survey suffered from varying amounts of non-responses. The section relating to pharmaceutical interventions had a significantly lower response rate relevant to other sections. This could be related to the fact that the questionnaire was sent to heads of radiotherapy departments, who were asked to forward it to an appropriate person responsible for smoking cessation within the trust. This indicates that smoking cessation services would benefit from a multidisciplinary approach to design and delivery. The survey was also only sent to a targeted audience of department managers, which may have introduced some bias if managers tried to put their departments in a more positive light. However, the high proportion of less positive responses suggests that this was not extensive.

## 6. Conclusions

This audit highlights that compliance with NICE smoking cessation guidance varies significantly between trusts. While beacons of good compliance with guidance exist, a number of trusts have adopted a pick-and-mix approach to the guidance that has been identified in this study.

Recommendations for future implementation and compliance to the NICE guidance include:

- A greater commitment from all trusts to fully comply with the smoke-free guidance and adhere to their policies. A key element of achieving this will be clear and consistent information and advice to patients about the benefits of quitting smoking or abstaining from smoking during their cancer treatment.
- A successful quit attempt or absence will be better supported by offering a full range of NRT products beyond transdermal patches. Practitioners should have access to other delivery methods such as quick mist sprays and inhalators that are quick acting pharmacotherapies via Patient Group Directive or as non-medical prescribers as well as access to non-nicotine-containing pharmaceuticals such as varenicline and bupropion.
- The creation of specific e-cigarette/vaping policies that make a distinction between conventional cigarettes and electronic devices.
- Practitioners and organisations have a responsibility to access free, and at the point of delivery, smoking cessation training delivered by the National Centre for Smoking Cessation training that includes all staff, from strategic management to non-clinical.
- A consistent electronic process and system for recording and monitoring smoking status and making referrals. It is essential that referrals are quick and easy to make and that progress and outcomes in each clinical area/department are monitored.
- Strong leadership and management to ensure secondary care premises (including grounds and vehicles) remain smoke-free.

## Figures and Tables

**Table 1:** Implementation of key National Institute Clinical Excellence (NICE) Guidance.

Item	Responses % (Number)		
Is your hospital site smoke-free?	No and currently no plans	Yes in theory	Yes in practice
	2.3% (1)	79.1% (34)	18.6% (8)
	No	Yes	
Is this policy enforced?	47.6% (20)	52.4% (22)	
Are e-cigarettes covered in your Trust’s smoking policy?	39.5% (15)	60.5% (23)	
Is the patient’s smoking status revisited periodically during active treatment?	68.4% (26)	31.6% (12)	
Are patients informed of the potential for increased acute side effects relating to smoking whilst undergoing cancer therapies?	2.7% (1)	97.3% (36)	
Are patients informed of the reduced effectiveness of cancer therapies if they smoke during treatment?	13.5% (5)	86.5% (32)	
Are patients advised/encouraged to abstain from smoking for a period of time prior to and after a radiotherapy fraction or administration of chemotherapy?	67.6% (25)	32.4% (12)	
What training is provided to front-line staff in smoking cessation services?	None	Brief Intervention Trainng	
	48.1% (13)	51.9% (14)	

**Table 2:** Enablers to achieve the smoke-free policy in practice.

Theme	Quotes
Designated tobacco control staff and supportive senior staff	“Designated tobacco control lead with responsibility for policy and compliance. One full-time and four part-time advisors (charity funded). Supportive chief executive and senior managers.”
	“Dedicated staff with specialist remit to promote the anti-smoking policy.”
Smoke-free signage and electronic messages	“Large signage at all entrances to the campus and throughout the campus. Large posters at doorways with dark red crosshatched areas to highlight non-smoking messages. Electronic messages relayed (smoke activated) at entrance buildings asking people to extinguish cigarettes.”
	“Signage, automatic announcements at entrances and areas where smokers would gather to announce that the site is smoke-free.”
Designated smoker areas	“Three smoking shelters are provided for those who have to smoke and that is the only place smoking is allowed by patients and visitors.”
Smoking cessation support	“Smoking cessation services are widely advertised.”
	“Smoking cessation clinic and support on site.”
	“Encouragement for anyone not to smoke, patients given patches, media involvement.“
Disciplinary action for staff breaches	“Disciplinary action for staff, patrols for people moving on.”
	“Policies to discipline smoking staff in uniform on site.”

**Table 3:** Barriers to achieving the smoke-free policy.

Theme	Quotes
Policy not enforced	“Patients and visitors disregard signs and no one stops them from smoking.”
	“There is an on-going tolerance/turning a blind eye to smokers standing outside buildings on-site.”
	“They (visitors) still sit on the benches outside smoking, even though they have no smoking signs on them! Essentially you can’t enforce a non-smoking area outside.”
	“It was enforced when it was first brought in.”
	“In theory all staff are asked to challenge smokers. In practice this doesn't happen.”
	“They started as totally smoke-free, but had to install smoking shelters away from the main entrance, as visitors were just ignoring it anyway.”
Staff concerns about enforcing policy	“Patients and visitors can become abusive if challenged.”
	“Staff cautious about challenging smokers in case of verbal abuse.”
	“It was driving the smokers to find somewhere to smoke covertly which became a greater risk.”
Policy breaches	“Ambulance/visiting health care staff smoking in uniform and undermining the policy.”
	“Patients and visitors smoking outside the main entrance was a challenge for a number of trusts. A couple of trusts erected or re-erected “smoking” shelters as a pragmatic approach, despite it being contrary to NICE guidance—although this did not always have the desired effect.”
	“The Trust had to relent and put up a smoking shelter for (patients and visitors), however, they rarely use it and still choose to smoke outside the main entrance. The smoking shelter is just across the road from the main entrance!”
	“Smokers disregard all enablers, it is impossible to enforce if people continue to believe that they can smoke outside. There are very few episodes where people smoke within the hospital but the grounds are much harder to police.”
Smoke-free signage	“Not having large signage—we only have small signs and A4 posters on sandwich boards.”
Patient choice	“Patients choice… staff are not prepared to challenge people as they are leaving work.”
	“Patients still wish to smoke… Freedom of individual.”

**Table 4:** Communication of the smoke-free policy and cessation support available for patients.

Item	*n*	Never	Rarely	Sometimes	Often	Always
Patients are provided with written and/or verbal information and advice about the smoke-free policy	40	22.5%	10.0%	17.5%	17.5%	32.5%
Information is provided about the different types of smoking cessation pharmacotherapies and types of behavioural support available	31	6.5%	12.9%	45.2%	19.4%	16.1%
The use of licensed nicotine-containing products for anyone who does not want to stop smoking completely is encouraged	28	7.1%	14.3%	50.0%	21.4%	7.1%
Licensed nicotine-containing products are offered to all patients who smoke as appropriate	26	15.4%	19.2%	30.8%	23.1%	11.5%
People who do not want to stop smoking completely are encouraged to use licensed nicotine-containing products to help reduce cravings to smoke during cancer therapies	23	13.0%	17.4%	26.1%	39.1%	4.3%
